# Opportunities for Enhanced Public Health Surveillance via Molecular Detection and Sequencing of Diverse Respiratory Viruses From Self-collected SARS-CoV-2 Antigen Test Swabs

**DOI:** 10.1093/ofid/ofae447

**Published:** 2024-08-10

**Authors:** Kat Schmidt, Simon D Pollett, Stephanie A Richard, Vivian Hogan, Emily Hone, Jennifer Rothenberg, Rezalina Tant, Michele Wayman, Chantele Friend, Kamala Thapa, Lola Ulomi, Julian Davies, Amber Michel, Timothy H Burgess, Robert J O’Connell, Mark P Simons, Drake H Tilley, Anthony C Fries, Rhonda E Colombo

**Affiliations:** Infectious Disease Clinical Research Program, Department of Preventive Medicine and Biostatistics, Uniformed Services University of the Health Sciences, Bethesda, Maryland, USA; The Henry M. Jackson Foundation for the Advancement of Military Medicine, Inc., Bethesda, Maryland, USA; Infectious Disease Clinical Research Program, Department of Preventive Medicine and Biostatistics, Uniformed Services University of the Health Sciences, Bethesda, Maryland, USA; The Henry M. Jackson Foundation for the Advancement of Military Medicine, Inc., Bethesda, Maryland, USA; Infectious Disease Clinical Research Program, Department of Preventive Medicine and Biostatistics, Uniformed Services University of the Health Sciences, Bethesda, Maryland, USA; The Henry M. Jackson Foundation for the Advancement of Military Medicine, Inc., Bethesda, Maryland, USA; Infectious Disease Clinical Research Program, Department of Preventive Medicine and Biostatistics, Uniformed Services University of the Health Sciences, Bethesda, Maryland, USA; The Henry M. Jackson Foundation for the Advancement of Military Medicine, Inc., Bethesda, Maryland, USA; Infectious Disease Clinical Research Program, Department of Preventive Medicine and Biostatistics, Uniformed Services University of the Health Sciences, Bethesda, Maryland, USA; The Henry M. Jackson Foundation for the Advancement of Military Medicine, Inc., Bethesda, Maryland, USA; Infectious Disease Clinical Research Program, Department of Preventive Medicine and Biostatistics, Uniformed Services University of the Health Sciences, Bethesda, Maryland, USA; The Henry M. Jackson Foundation for the Advancement of Military Medicine, Inc., Bethesda, Maryland, USA; Infectious Disease Clinical Research Program, Department of Preventive Medicine and Biostatistics, Uniformed Services University of the Health Sciences, Bethesda, Maryland, USA; The Henry M. Jackson Foundation for the Advancement of Military Medicine, Inc., Bethesda, Maryland, USA; Infectious Disease Clinical Research Program, Department of Preventive Medicine and Biostatistics, Uniformed Services University of the Health Sciences, Bethesda, Maryland, USA; The Henry M. Jackson Foundation for the Advancement of Military Medicine, Inc., Bethesda, Maryland, USA; Infectious Disease Clinical Research Program, Department of Preventive Medicine and Biostatistics, Uniformed Services University of the Health Sciences, Bethesda, Maryland, USA; The Henry M. Jackson Foundation for the Advancement of Military Medicine, Inc., Bethesda, Maryland, USA; Infectious Disease Clinical Research Program, Department of Preventive Medicine and Biostatistics, Uniformed Services University of the Health Sciences, Bethesda, Maryland, USA; The Henry M. Jackson Foundation for the Advancement of Military Medicine, Inc., Bethesda, Maryland, USA; Infectious Disease Clinical Research Program, Department of Preventive Medicine and Biostatistics, Uniformed Services University of the Health Sciences, Bethesda, Maryland, USA; The Henry M. Jackson Foundation for the Advancement of Military Medicine, Inc., Bethesda, Maryland, USA; Infectious Disease Clinical Research Program, Department of Preventive Medicine and Biostatistics, Uniformed Services University of the Health Sciences, Bethesda, Maryland, USA; The Henry M. Jackson Foundation for the Advancement of Military Medicine, Inc., Bethesda, Maryland, USA; Infectious Disease Clinical Research Program, Department of Preventive Medicine and Biostatistics, Uniformed Services University of the Health Sciences, Bethesda, Maryland, USA; The Henry M. Jackson Foundation for the Advancement of Military Medicine, Inc., Bethesda, Maryland, USA; Infectious Disease Clinical Research Program, Department of Preventive Medicine and Biostatistics, Uniformed Services University of the Health Sciences, Bethesda, Maryland, USA; Infectious Disease Clinical Research Program, Department of Preventive Medicine and Biostatistics, Uniformed Services University of the Health Sciences, Bethesda, Maryland, USA; Infectious Disease Clinical Research Program, Department of Preventive Medicine and Biostatistics, Uniformed Services University of the Health Sciences, Bethesda, Maryland, USA; Naval Health Clinic Annapolis, Annapolis, Maryland, USA; United States Air Force School of Aerospace Medicine (USAFSAM), Wright-Patterson Air Force Base, Ohio, USA; Infectious Disease Clinical Research Program, Department of Preventive Medicine and Biostatistics, Uniformed Services University of the Health Sciences, Bethesda, Maryland, USA; The Henry M. Jackson Foundation for the Advancement of Military Medicine, Inc., Bethesda, Maryland, USA

**Keywords:** human influenza, public health surveillance, respiratory tract infection, SARS-CoV-2, whole-genome sequencing

## Abstract

We sequenced and genotyped severe acute respiratory syndrome coronavirus 2 (SARS-CoV-2), influenza, adenovirus, and respiratory syncytial virus, among other pathogens, from residual anterior nasal swabs self-collected for rapid SARS-CoV-2 antigen testing at the US Naval Academy. This is a key proof-of-concept for an acute respiratory infection surveillance approach, which could leverage prevalent SARS-CoV-2 antigen self-testing.

Acute respiratory infections (ARIs) are a major source of morbidity and mortality [1], and molecular ARI surveillance is a key public health tool [2] which is particularly important in congregate settings such as university campuses [3, 4], skilled nursing facilities [5], and military settings (eg, aircraft carriers, academies, etc.) [6]. For example, among US military recruits, ∼23% of medical encounters were attributed to ARIs in 2019, making ARIs a major cause of morbidity and lost duty days [7].

For clinical diagnosis of ARIs, self-collected severe acute respiratory syndrome coronavirus 2 (SARS-CoV-2) rapid antigen (Ag) tests have become increasingly prevalent, partially because of their fast turnaround time and lower cost. Conversely, clinically collected nasopharyngeal (NP) swab polymerase chain reaction (PCR) testing for SARS-CoV-2 has declined [8]. The decline in NP swab testing for coronavirus disease 2019 (COVID-19)–like illness has reduced the availability of specimens which could contribute important molecular surveillance data for SARS-CoV-2 and other respiratory viruses. While widely used, SARS-CoV-2 rapid Ag tests do not provide infecting variant details, may fail to detect some SARS-CoV-2 variants entirely, and may be negative during early COVID-19 illness [9]. Residual nasal swab specimens from Ag tests are not routinely used for sequencing or the detection of other respiratory viruses. These drawbacks create gaps in public health surveillance that could impact patient care and population mitigation strategies, such as limiting surveillance for vaccine- and/or treatment-resistant strains.

Early data support the use of self-collected SARS-CoV-2 Ag tests to augment genomic surveillance of SARS-CoV-2 and other respiratory viruses. Nazario-Toole et al. pioneered the technique of performing whole-genome sequencing (WGS) for SARS-CoV-2 on residual anterior nasal swabs used in rapid Ag testing (“Ag swabs”) [10]. Other studies have noted the feasibility of WGS of influenza and respiratory syncytial virus (RSV) from residual SARS-CoV-2 Ag swabs [11, 12]. Overall, there has been limited research into the detection and sequencing of a broader range of respiratory viruses from self-collected SARS-CoV-2 Ag tests.

To address this gap, we leveraged an epidemiological study of medically attended ARIs (MAARIs) at the US Naval Academy (USNA; Annapolis, MD, USA) to demonstrate the feasibility and validity of PCR detection and sequencing of a variety of respiratory viruses, including SARS-CoV-2, influenza, adenovirus, and RSV, from residual self-collected SARS-CoV-2 rapid Ag tests.

## METHODS

Acute Respiratory Infections at the Academy (“ARIA”) is an observational, augmented surveillance study of MAARIs at the USNA. All data and specimens are obtained for clinical purposes, and there is no direct research interaction with human subjects. The surveillance population is comprised of personnel assigned to USNA who presented to the Brigade Medical Unit (BMU) for an ARI and/or provided a respiratory specimen. The study protocol was approved by the USUHS Institutional Review Board in compliance with all applicable federal research data regulations governing the protection of human subjects as prescribed in 45 CFR 46.

The study procedures are summarized in Supplementary Figure 1. Briefly, per standard of care (SOC), all patients presenting to the BMU with ARI symptoms underwent SARS-CoV-2 rapid Ag testing using self-collected nasal swabs. If that Ag test was negative, an NP swab was also collected (by a clinical provider) and tested on-site with a real-time reverse transcription PCR (rRT-PCR) for SARS-CoV-2, influenza, and RSV (GeneXpert Xpress CoV-2/Flu/RSV plus, Cepheid, Sunnyvale, CA, USA). All NP swabs included in this analysis had a corresponding negative SARS-CoV-2 Ag test, except 1 case in which the Ag test was positive.

After SOC testing, rapid Ag swabs were placed into DNA/RNA Shield (Zymo Research, Irvine, CA, USA), and NP swabs remained in the residual viral transport media used for SOC testing (see the Supplementary Data for additional details). Swabs were then stored at −80°C and shipped weekly to the United States Air Force School of Aerospace Medicine (USAFSAM). At USAFSAM, all residual nasal and NP swabs underwent testing via the Centers for Disease Control and Prevention (CDC) Influenza SARS-CoV-2 (FluSC2) Multiplex Assay (https://archive.cdc.gov/www_cdc_gov/coronavirus/2019–ncov/lab/multiplex.html) [13–15]. Residual NP swabs also underwent an expanded multiplex PCR for additional respiratory pathogen testing (NxTAG Respiratory Pathogen Panel [RPP], Diasorin/Luminex, Austin, TX, USA). WGS was performed on nasal Ag and NP PCR swabs with a CDC FluSC2 cycle threshold (Ct) value ≤36 for SARS-CoV-2, influenza A, or influenza B; WGS was also performed on NP swabs that were RPP positive for either adenovirus or RSV and had a Ct value ≤36 in subsequent virus-specific rRT-PCR testing (Supplementary Data).

## RESULTS

In the first 5 months of the study (3/20/23–8/31/23), 1200 individuals presented at the BMU for a total of 1337 MAARIs (Supplementary Table 1). For 98.2% (n = 1313) of MAARIs, ≥1 respiratory specimen was collected. At least 1 respiratory virus was detected in 44% of MAARIs (n = 592); the most common viruses identified were rhino/enterovirus and SARS-CoV-2 (Supplementary Figure 2).

During this time, 98 Ag swabs had SARS-CoV-2 or influenza detected on FluSC2 rRT-PCR at USAFSAM. Additionally, when adenovirus or RSV was detected from an NP swab via RPP, we performed a specific rRT-PCR assay for either RSV or adenovirus, as applicable, on the corresponding Ag swab for that case. This resulted in 104 total Ag swabs positive for either adenovirus (n = 4), influenza (n = 5), RSV (n = 2), or SARS-CoV-2 (n = 93). Amplicon sequencing was attempted on all Ag swabs with Ct ≤36 and sufficient remaining sample volume (n = 90 attempted; 86.5%). Viral WGS was successful for 79 (87.8%) of these Ag swabs (Table 1). We identified 2 adenovirus types (C1 and E4; n = 1 and n = 1), influenza B virus (clade V1A.3a.2; n = 2), influenza A/H1N1pdm09 virus (clade 6B.1A.5a.2a.1; n = 1), an RSV strain (B6, genotype GB5.0.5a; n = 2), and numerous SARS-CoV-2 Omicron lineages (Table 1). Notably, we successfully sequenced SARS-CoV-2 from several Ag swabs that had low concentrations of SARS-CoV-2 viral RNA (eg, 7.6 viral genome equivalents/reaction; PCR Ct 34.0—XBB.2.3; 8.5 viral genome equivalents/reaction; PCR Ct 35.0—XBB.1.5).

**Table 1.** Genotyping Results for All Residual Antigen Swabs With a Pathogen of Interest Detected at USAFSAM (03/20–08/31/2023)^a^

Virus, Genotype	No. (%)
**Adenovirus (n = 4)**	
Adenovirus C1	1 (25)
Adenovirus E4	1 (25)
Not attempted^b^	2 (50)
**Influenza (n = 5)**	
V1A.3a.2 (B-Victoria)	2 (40)
6B.1A.5a.2a.1 (A/H1N1)	1 (20)
Not attempted^c^	1 (20)
Attempted and unsuccessful^d^	1 (20)
**RSV (n = 2)**	
B6; GB5.0.5a	2 (100)
**SARS-CoV-2 (n = 93)** ^e^	
CH.1.1.21	1 (1.1)
EG.5.1	1 (1.1)
FD.1.1	9 (9.7)
HV.1	1 (1.1)
XBB.1	1 (1.1)
XBB.1.16.2	20 (21.5)
XBB.1.19.1	1 (1.1)
XBB.1.42.2	1 (1.1)
XBB.1.5	7 (7.5)
XBB.1.5.10	6 (6.5)
XBB.1.5.59	1 (1.1)
XBB.1.9.1	1 (1.1)
XBB.1.9.2	1 (1.1)
XBB.2.3	19 (20.4)
XBB.2.3.13	1 (1.1)
XBB.2.3.5	1 (1.1)
Not attempted^b^	11 (11.8)
Attempted and unsuccessful^f^	10 (10.8)

Abbreviations: Ag, antigen; Ct, cycle threshold; PCR, polymerase chain reaction; RSV, respiratory syncytial virus; SARS-CoV-2, severe acute respiratory syndrome coronavirus 2; USAFSAM, United States Air Force School of Aerospace Medicine.

^a^Sequencing was attempted on swabs where the Ct was ≤36 and there was sufficient sample remaining (86.5% of positive Ag swabs). Additional sequencing results by PCR not shown here.

^b^Sequencing not attempted due to high Ct.

^c^Re-extract was negative for flu, unable to attempt sequencing.

^d^Specimen had high CT (33.8) and was subtyped as B-Victoria using a b-lineage assay.

^e^Includes an antigen swab with 7.6 viral genome equivalents/reaction and another antigen swab with 8.5 viral genome equivalents/reaction.

^f^SARS-CoV-2 Ct >27 for all sequence failures.

A subset of residual Ag swabs was selected for further evaluation with RPP (Supplementary Figure 1, tan boxes). Briefly, we identified individuals who had paired NP and Ag swabs from their initial encounter, where the NP swabs were positive for any virus by RPP and the Ag swabs had sufficient sample remaining to perform RPP testing. This resulted in 58 Ag swabs being additionally tested with RPP; on the Ag swabs, we found 56 of the 63 viruses originally identified on the NP swabs, plus an adenovirus infection that was missed on the paired NP swab (Table 2). Sensitivity was 89.7%, and Cohen's (unweighted) Kappa was 0.85 (95% CI, 0.75–0.95), which indicated strong agreement between residual nasal Ag and NP swabs.

**Table 2.** Respiratory Pathogen Panel Results for Paired Residual Nasal Antigen Swabs and Nasopharyngeal PCR Swabs (03/20–08/31/2023)^a,b^

	Swab Type	
	NP PCR (n = 58)	Nasal Ag (n = 58)
Rhinovirus/enterovirus	24	22
Seasonal coronavirus	20	18
Human metapneumovirus	9	8
Parainfluenza	5	3
Influenza	3	3
SARS-CoV-2	2	2
Adenovirus	0	1^b^
RSV	0	0
Bocavirus	0	0
No virus detected	0	5
Total number of viruses detected	63	57

Abbreviations: Ag, antigen; Ct, cycle threshold, NP, nasopharyngeal; PCR, polymerase chain reaction; Rp, human RNase P; RPP, Respiratory Pathogen Panel; RSV, respiratory syncytial virus.

^a^Fifty-eight individuals with NP swabs that were positive on RPP for ≥1 virus were chosen for an RPP pilot study on Ag swabs. Among the 58 Ag swabs, we detected ≥1 virus on 53 swabs (91.4%). On Ag swabs, 56/63 viruses detected on NP swabs were also detected (88.9%). Overall agreement by Cohen's Kappa was 0.85.

^b^For 1 person, the NP swab tested positive for rhinovirus/enterovirus only, but their paired Ag swab tested positive for both rhinovirus/enterovirus and adenovirus (NP Rp Ct, 26.5; nasal Ag Rp Ct, 28.8; nasal Ag adenovirus Ct, 40.8).

Lastly, we used the Twist Comprehensive Viral Research Panel (Twist Bioscience, San Francisco, CA, USA) to determine if sequencing of nonamplified target pathogens—as well as sequencing of coinfections—was feasible from Ag swabs. We selected a set of 18 paired NP and nasal Ag swabs (Supplementary Figure 1, gray boxes) to perform hybrid enrichment sequencing and compare sequencing sensitivity results. NP and nasal Ag swab results matched for 17/18 pairs (94.4%) (Supplementary Table 2). Shared nucleotide identity comparison of consensus genomes from the Twist Comprehensive Viral Panel was similar to that using the standard amplicon-based viral WGS from Ag and NP swabs (Supplementary Tables 3 and 4).

## DISCUSSION

We have demonstrated the potential validity of multiplex PCR, sequencing, and genotyping of multiple respiratory pathogens from a residual anterior nasal swab repurposed after use in rapid SARS-CoV-2 Ag testing. Furthermore, we have shown the ability to determine SARS-CoV-2 variants (and genotypes for other respiratory viruses) from Ag swabs with very low quantities of genetic material. Our results indicate that residual self-collected rapid Ag tests may offer incremental epidemiological information and may prompt simpler acute respiratory surveillance diagnostic algorithms, which can, in part, leverage self-collected rapid Ag test swabs in lieu of more resource-intensive NP swab collection by trained staff. This may be particularly valuable in settings where cost and/or infrastructure considerations limit the availability of point-of-care nucleic acid amplification testing.

This study has limitations. The study period encompassed just over 5 months, and only 7 specimens with non-SARS-CoV-2 respiratory viruses were sequenced and typed. Nevertheless, these pilot data on the successful use of residual Ag swabs for ARI surveillance provide key technical proof-of-concept for use in genomic epidemiology, especially as rapid Ag testing is increasingly used for SARS-CoV-2 diagnosis. These results support potentially significant opportunities for public health genomic surveillance of SARS-CoV-2 and other respiratory viruses. Future studies should examine the yield of molecular detection and sequencing of an even broader range of respiratory viruses from residual Ag tests, including Ag swabs that are stored and/or mailed at ambient temperatures, to facilitate potentially very broad community-based genomic surveillance.

## Supplementary Material

ofae447_Supplementary_Data

## Supplementary Data

Supplementary materials are available at *Open Forum Infectious Diseases* online. Consisting of data provided by the authors to benefit the reader, the posted materials are not copyedited and are the sole responsibility of the authors, so questions or comments should be addressed to the corresponding author.
