# Supplementary material for: Opportunities for Enhanced Public Health Surveillance via Molecular Detection and Sequencing of Diverse Respiratory Viruses From Self-collected SARS-CoV-2 Antigen Test Swabs
Source: Open Forum Infect Dis. 2024 Aug 10;11(8):ofae447. doi: 10.1093/ofid/ofae447 (PMC11339864; doi:10.1093/ofid/ofae447)
Supplement: ofae447_Supplementary_Data [file ofae447_supplementary_data.zip › Schmidt_Colombo_ARIA_Supplemental_Material_Revised_clean.docx]

**Supplemental Material**

**Supplemental Methods**

The Brigade Medical Unit (BMU) primarily used the BinaxNow COVID-19 Antigen Card Tests with the Puritan Sterile Foam Tipped Applicator (n=89; 85.6%) (Abbott Diagnostics Scarborough, Inc.) for rapid SARS-CoV-2 testing during the 5 months covered by this analysis. During one month – when there was a shortage of BinaxNow test kits at the BMU – QuickVue At Home OTC COVID-19 Tests with individually wrapped sterile foam swabs were substituted (n=15; 14.4%).

Midshipmen presenting to the clinic with symptoms concerning for possible COVID-19 were instructed by clinic staff to self-collect an anterior nasal swab (using the nasal swab contained in the antigen test kit being used by the clinic at that time), and clinic personnel processed the swab and read the result according to standard practices for that test kit [https://www.fda.gov/media/141570/download, BinaxNow instructions for use; https://www.fda.gov/media/147265/download, QuickVue instructions for use]. Each residual swab was placed in a biohazard bag in a designated study bin for a member of the study team to retrieve (daily). In the cases where a BinaxNow test kit was used, the nasal swab was left inside the test card then placed within the biohazard bag; for the QuickVue tests, only the residual swab was retained and placed in the biohazard bag.

After retrieving the residual nasal swab, the research laboratory technician (working within a Biological Safety Cabinet and wearing appropriate personal protective equipment) removed the nasal swab from biosafety bag (and test card, as applicable), cut the nasal swab tip at the scored line, placed the swab tip into a cryotube containing 1mL of specialized transport media (Zymo DNA/RNA Shield), and labeled the tube with a study specific label containing a specimen ID and coded subject ID.

We performed amplicon sequencing on SARS-CoV-2, influenza, adenovirus, and RSV positive specimens identified from nasopharyngeal (NP) or rapid antigen (Ag) swabs. We used either the midnight V2 or Artic V5 primer approaches for amplification of SARS-CoV-2 [1]. We utilized a universal primer sequencing approach for influenza A and for influenza B viruses [2-3]. For whole genome adenovirus amplification, we used species-specific tiling approaches designed by Joshua Quick (https://github.com/quick-lab/HAdV). For RSV, amplicon tiles were designed for each RSV type, A and B, by Daniel Maloney (https://github.com/artic-network/artic-rsv). Nextera XT library preparation kits (Illumina, San Diego, CA) were used to prepare amplified products for sequencing and sequencing runs were performed on an Illumina NextSeq 550.

Analysis of amplicon sequencing data was performed with pathogen specific analysis pipelines consistent with routine workflows at the United States Air Force School of Aerospace Medicine. For SARS-CoV-2, we utilized the Mad River Workflow (https://github.com/usafsam/mad_river_wf), which is a workflow derived from the Cecret Workflow (https://github.com/UPHL-BioNGS/Cecret). For influenza, we used a standard deployment of the Iterative Refinement Meta Assembler (IRMA) [4]. For adenovirus and RSV we constructed modules specific to both pathogens within the IRMA software.

For pathogens for which we did not have a pathogen specific amplicon tiling scheme designed, we used the Twist Comprehensive Viral panel (Twist Bioscience, San Francisco, CA). Both NP and Ag swabs were processed similarly. Sequencing libraries were run on an Illumina NextSeq 550. For Twist data analyses, we used the Kraken2 software [5] to initially identify the presence or absence of the target pathogens that were originally identified on these specimens on either the Luminex NxTAG Respiratory Pathogen Panel or the CDC FluSC2 assay. We then used a modified version of the Mad River Workflow and performed reference-based assembly for the target pathogen. RefSeq genomes for human metapneumovirus, parainfluenza 2, human coronavirus HKU1, 229E, NL63, and OC43 were used.

Once consensus genomes were obtained for each RPP and or FluSC2 positive target pathogen, we measured the percent nucleotide identity between Ag and NP swabs from the same individual using the Molecular Evolutionary Genetics Analysis software [6]. We compared nucleotide identity at locations where we had at least 10 reads for a specific base on both the NP and Ag swab sequence. Therefore, a nucleotide was only compared if it was present in both sequences and either conserved or variable. All sequencing FASTA data have been submitted to NCBI GenBank.

Of note, we did observe the presence of SARS-CoV-2 sequencing reads when initially run through Kraken2 on specimens that were not originally identified as having SARS-CoV-2 on any multiplex PCR testing. Twist sequence data were at levels that were indicative of positives in other specimens. The variability observed in those SARS-CoV-2 genomes were not indicative of contamination but were too poor of reconstructions to indicate why they were not identified through multiplex PCR.

**Supplemental References**

1. Freed NE, Vlková M, Faisal MB, Silander OK. Rapid and inexpensive whole-genome sequencing of SARS-CoV-2 using 1200 bp tiled amplicons and Oxford Nanopore Rapid Barcoding. Biol Methods Protoc. 2020;5(1):bpaa014. Published 2020 Jul 18. doi:10.1093/biomethods/bpaa014
2. Zhou B, Donnelly ME, Scholes DT, et al. Single-reaction genomic amplification accelerates sequencing and vaccine production for classical and Swine origin human influenza A viruses. J Virol. 2009;83(19):10309-10313. doi:10.1128/JVI.01109-09
3. Zhou B, Lin X, Wang W, et al. Universal influenza B virus genomic amplification facilitates sequencing, diagnostics, and reverse genetics. J Clin Microbiol. 2014;52(5):1330-1337. doi:10.1128/JCM.03265-13
4. Shepard SS, Meno S, Bahl J, Wilson MM, Barnes J, Neuhaus E. Viral deep sequencing needs an adaptive approach: IRMA, the iterative refinement meta-assembler [published correction appears in BMC Genomics. 2016 Oct 13;17 (1):801]. BMC Genomics. 2016;17(1):708. Published 2016 Sep 5. doi:10.1186/s12864-016-3030-6
5. Wood DE, Lu J, Langmead B. Improved metagenomic analysis with Kraken 2. Genome Biol. 2019;20(1):257. Published 2019 Nov 28. doi:10.1186/s13059-019-1891-0
6. Tamura K, Stecher G, Peterson D, Filipski A, Kumar S. MEGA6: Molecular Evolutionary Genetics Analysis version 6.0. Mol Biol Evol. 2013;30(12):2725-2729. doi:10.1093/molbev/mst197

**Supplemental Figures**

**Figure S1. Specimen Collection and Processing for ARIA.**


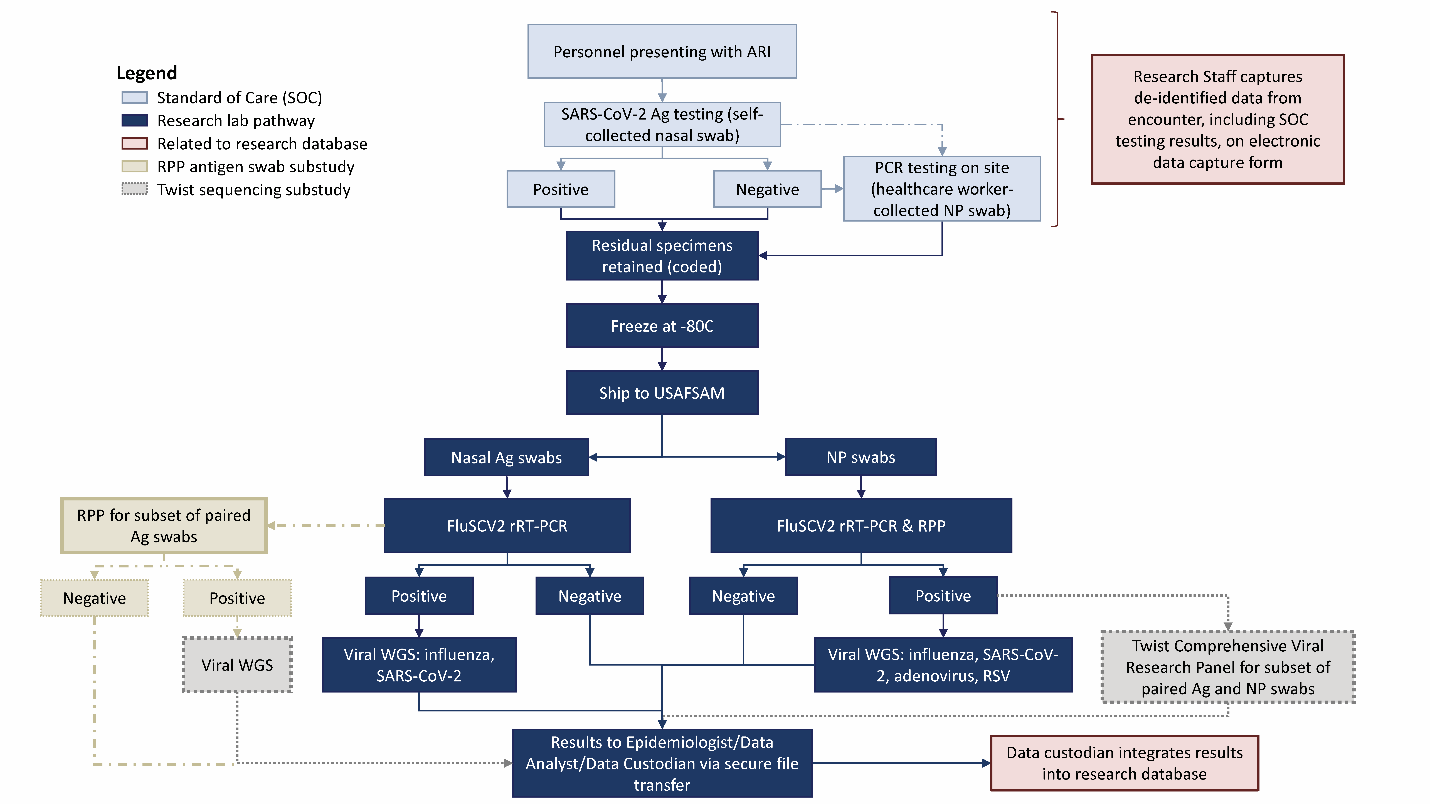


Flowchart showing respiratory specimen collection and processing for ARIA at the US Naval Academy and United States Air Force School of Aerospace Medicine (USAFSAM). Samples include self-collected nasal swabs for SARS-CoV-2 antigen testing and/or healthcare worker-collected nasopharyngeal swabs for RT-PCR testing. Abbreviations: Acute Respiratory Infection (ARI); antigen (Ag); Standard of Care (SOC); real-time reverse-transcription polymerase chain reaction (rRT-PCR); nasopharyngeal (NP); respiratory syncytial virus (RSV); NxTAG Respiratory Pathogen Panel (RPP); whole genome sequencing (WGS).

**Figure S2. Viruses detected at the US Naval Academy via ARIA.**


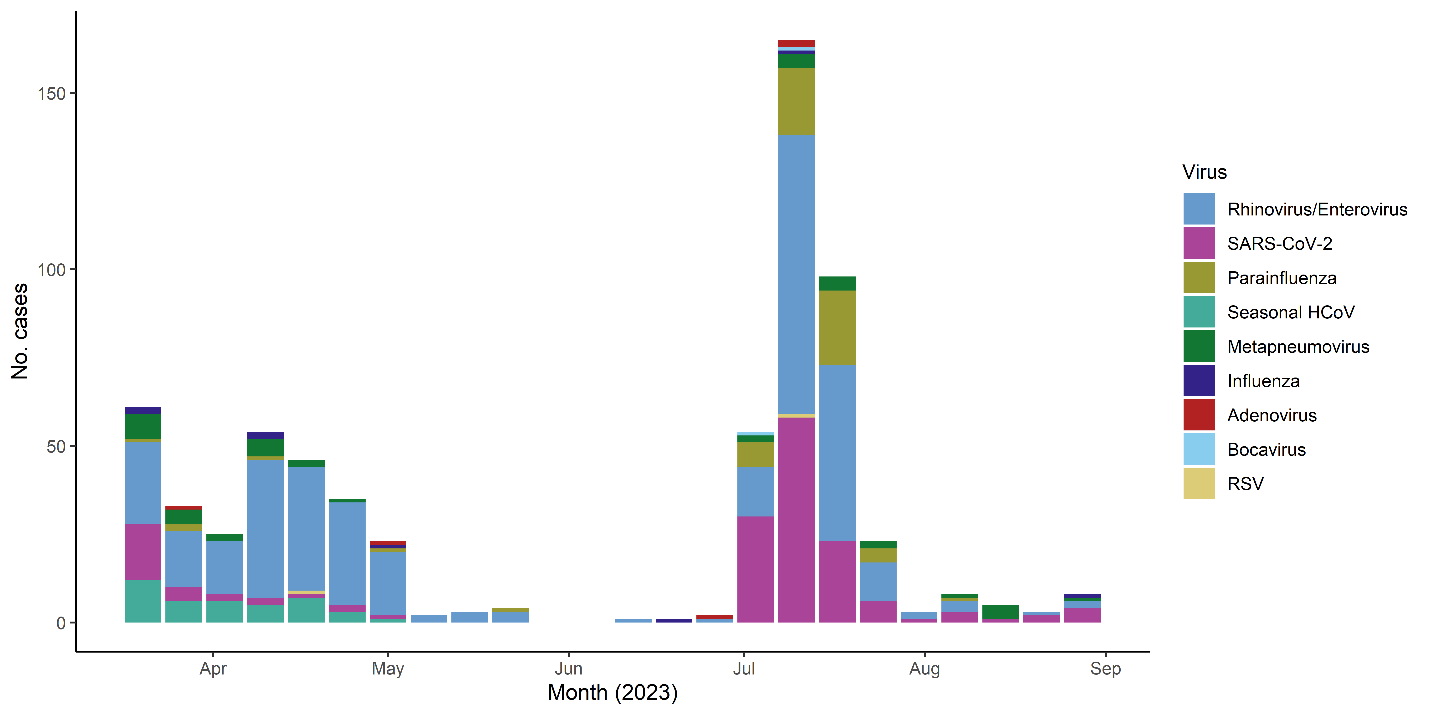
From March 20^th^ – August 31^st^, 2023, viruses were detected in 592 (44%) of all medically-attended acute respiratory infections (MAARIs). For those 592 MAARIs, 657 viruses were either identified via standard of care (SOC) (n=141; 21.5%) or further molecular testing at USAFSAM (n=516; 78.5%); viruses detected on multiple swabs collected for the same illness were only counted once per MAARI. SOC testing missed 22 cases (13.5%) of SARS-CoV-2, two cases (25%) of influenza, and one case (50%) of RSV, which were detected by enhanced molecular testing at USAFSAM. Regarding NP swabs specifically, SOC CoV-2/Flu/RSV rRT-PCR results had high concordance with the Flu/SC2 rRT-PCR and RPP (SARS-CoV-2: 98.36% concordance with both; influenza 100% concordance with both; RSV: 99.89% concordance with RPP). There were 60 cases of co-infections; the most common co-infection category was SARS-CoV-2 and rhino/enterovirus (n=16; 26.7%), followed by parainfluenza and rhino/enterovirus (n=13; 21.7%).

**Supplemental Tables**

| **Table S1.** Characteristics of individuals presenting to the clinic with acute respiratory illness (03/20 – 08/31/2023): overall and respiratory pathogen panel pilot subset | | |
| --- | --- | --- |
|  | **Overall** | **Expanded multiplex PCR pilot study** |
|  | **(n = 1200)** | **(n = 58)** |
| Age (years), median (IQR) | 19.0 (18.0, 21.0) | 21.0 (20.0, 22.0) |
| Sex, n (%) |  |  |
| Male | 764 (63.7%) | 36 (62.1%) |
| Female | 436 (36.3%) | 22 (37.9%) |
| Race/Ethnicity, n (%) |  |  |
| White, Non-Hispanic | 618 (51.5%) | 34 (59.4%) |
| Unknown/Not Reported | 158 (13.2%) | 11 (19.0%) |
| Asian, Non-Hispanic | 152 (12.7%) | 4 (6.9%) |
| Hispanic or Latino | 115 (9.6%) | 2 (3.4%) |
| Black, Non-Hispanic | 103 (8.6%) | 5 (8.6%) |
| Other, Non-Hispanic | 54 (4.5%) | 2 (3.4%) |
| Personnel Status, n (%) |  |  |
| Midshipmen | 1,180 (98.3%) | 58 (100.0%) |
| Commandant Staff | 20 (1.7%) | 0 (0.0%) |

| **Table S2.** Comparison of respiratory pathogen panel and Kraken2 output from Twist Comprehensive Viral panel run on rapid antigen and nasopharyngeal swabs^a^ | | | |
| --- | --- | --- | --- |
| **Individual** | **Platform** | **Swab type** | |
|  |  | **Nasal Ag** | **NP PCR** |
| Person #1 | RPP | SARS-CoV-2 | SARS-CoV-2 |
|  | Kraken2 | SARS-CoV-2 | SARS-CoV-2 |
| Person #2 | RPP | Influenza A H1 | Influenza A H1 |
|  | Kraken2 | Influenza A H1 | Influenza A H1^b^ |
| Person #3 | RPP | Human coronavirus NL63 | Human coronavirus NL63 |
|  | Kraken2 | Human coronavirus NL63 | Human coronavirus NL63 |
| Person #4 | RPP | Human coronavirus OC43 | Human coronavirus OC43 |
|  | Kraken2 | Human coronavirus OC43 | Human coronavirus OC43 |
| Person #5 | RPP | Human metapneumovirus | Human metapneumovirus |
|  | Kraken2 | Human metapneumovirus | Human metapneumovirus |
| Person #6 | RPP | Parainfluenza 2 | Parainfluenza 2 |
|  | Kraken2 | Parainfluenza 2^b^ | Parainfluenza 2^b^ |
| Person #7 | RPP | Rhinovirus/enterovirus; SARS-CoV-2 | Rhinovirus/enterovirus; SARS-CoV-2 |
|  |  |  |  |
|  | Kraken2 | Human rhinovirus NAT001; SARS-CoV-2 | Human rhinovirus NAT001; SARS-CoV-2 |
| Person #8 | RPP | Human coronavirus 229E | Human coronavirus 229E |
|  | Kraken2 | Human coronavirus 229E^b^ | Human coronavirus 229E |
| Person #9 | RPP | Human coronavirus 229E; Human metapneumovirus | Human coronavirus 229E; Human metapneumovirus |
|  |  |  |  |
|  | Kraken2 | Human coronavirus 229E^b^; Human metapneumovirus^b^ | Human coronavirus 229E^b^; Human metapneumovirus^b^ |
| Person #10 | RPP | Influenza B | Influenza B |
|  | Kraken2 | Influenza B (B/Lee/1940)^b^ | Influenza B (B/Lee/1940) |
| Person #11 | RPP | Parainfluenza 2 | Parainfluenza 2 |
|  | Kraken2 | Parainfluenza 2^b^ | Negative^c^ |
| Person #12 | RPP | Human metapneumovirus | Human metapneumovirus |
|  | Kraken2 | Negative^b,c,d^ | Negative^c,d^ |
| Person #13 | RPP | Rhinovirus/enterovirus | Rhinovirus/enterovirus |
|  | Kraken2 | Rhinovirus A1 | Negative^c^ |
| Person #14 | RPP | Human coronavirus HKU1 | Human coronavirus HKU1 |
|  | Kraken2 | Human coronavirus HKU1 | Human coronavirus HKU1 |
| Person #15 | RPP | Human coronavirus HKU1; Rhinovirus/enterovirus | Human coronavirus HKU1; Rhinovirus/enterovirus |
|  | Kraken2 | Human coronavirus HKU1^b^; Parainfluenza 1^b^ | Human coronavirus HKU1; *No parainfluenza 1* |
| Person #16 | RPP | Human metapneumovirus | Human metapneumovirus |
|  | Kraken2 | Human metapneumovirus^b^ | Human metapneumovirus |
| Person #17 | RPP | Rhinovirus/enterovirus | Rhinovirus/enterovirus |
|  | Kraken2 | Human rhinovirus NAT001 | Human rhinovirus NAT001 |
| Person #18 | RPP | Influenza B | Influenza B |
|  | Kraken2 | Influenza B (B/Lee/1940) | Influenza B (B/Lee/1940) |
| *^a^Abbreviations: respiratory pathogen panel (RPP); antigen (Ag); nasopharyngeal (NP).*  *^b^These specimens had detection of SARS-CoV-2 reads in the Twist sequencing data.*  *^c^A consensus genome was obtained for the expected pathogen despite an initial indication of a negative result from Kraken2.*  *^d^Positive control for human metapneumovirus had failed to be found in this sequencing run as well, indicating the potential for laboratory error.* | | | |
|  | | | |

| **Table S3.** Nucleotide identity comparison of consensus genomes from Twist Comprehensive Viral panel | | | |
| --- | --- | --- | --- |
| **Individual** | **Virus** | **Shared % identity (no. of bases that differ)** | **No. of bases covered^a^** |
| Person #3 | Human coronavirus NL63 | 100% | 27,497 |
| Person #4 | Human coronavirus OC43 | 100% | 30,447 |
| Person #5 | Human metapneumovirus | 100% | 13,011 |
| Person #6 | Parainfluenza virus 2 | 100% | 15,581 |
| Person #7 | Rhinovirus/enterovirus | 99.1% (39) | 4,429 |
| Person #8 | Human coronavirus 229E | 99.9% (2) | 27,230 |
| Person #9^b^ | Human coronavirus 229E | n/a | n/a |
| Person #9 | Human metapneumovirus | 100% | 12,982 |
| Person #11 | Parainfluenza virus 2 | 100% | 15,477 |
| Person #12^c^ | Human metapneumovirus | n/a | n/a |
| Person #13 | Rhinovirus/enterovirus | 100% | 6,129 |
| Person #14 | Human coronavirus HKU1 | 100% | 29,638 |
| Person #15 | Human coronavirus HKU1 | 100% | 29,515 |
| Person #15 | Rhinovirus/enterovirus^d^ | 100% | 2,681 |
| Person #16 | Human metapneumovirus | 100% | 12,913 |
| Person #17 | Rhinovirus/enterovirus | 100% | 7,131 |
| *^a^Full expected genome lengths are as follows: coronavirus NL63 = 27,553; coronavirus OC43 = 30,741; coronavirus 229E = 27,317; coronavirus HKU1 = 29,926; human metapneumovirus = 13,350; parainfluenza virus 2 = 15,646; rhinovirus NAT001 = 6,944; rhinovirus A1 = 7,137.*  *^b^Antigen swab sequencing failed to identify any human coronavirus 229E reads in this preparation.*  *^c^Both the nasal Ag and NP PCR swabs failed to recover human metapneumovirus for this individual. Of note, the positive control for human metapneumovirus also failed on this library prep and could indicate an artifact.*  *^d^For this individual, the respiratory pathogen panel only found a positive rhino/enterovirus result on the rapid Ag swab.* | | | |

| **Table S4.** Nucleotide identity comparison of amplicon tiling protocols between nasopharyngeal and nasal rapid antigen swabs | | | |  |
| --- | --- | --- | --- | --- |
| **Virus** | **Individual** | **Shared % identity (no. of bases that differ)** | **No. of bases covered^a^** | |
| SARS-CoV-2 | Person #1 | 99.99% (1) | 25,899 | |
| SARS-CoV-2 | Person #7 | Failed | n/a | |
| SARS-CoV-2 | Person #21 | 100.00% | 3,330 | |
| SARS-CoV-2 | Person #22 | 99.99% (1) | 26,881 | |
| SARS-CoV-2 | Person #23 | 100% | 28,908 | |
| SARS-CoV-2 | Person #25^b^ | 68.32% (83) | 262 | |
| SARS-CoV-2 | Person #26 | 100.00% | 27,107 | |
| Influenza A | Person #2^c^ | 100.00% | 1,701 | |
| Influenza B | Person #10^c^ | 100.00% | 1,749 | |
| Influenza B | Person #18^c^ | 100.00% | 1,749 | |
| RSV-B | Person #24 | 100.00% | 15,191 | |
| RSV-B | Person #27 | 99.98% (3) | 15,156 | |
| Adenovirus | Person #19 | 100.00% | 35,778 | |
| Adenovirus | Person #20^d^ | 99.94% (12) | 21,279 | |
| *^a^Full expected genome lengths are as follows: SARS-CoV-2 = 29,903; Influenza A (HA) = 1701; Influenza B (HA) = 1749; RSV-B = 15,191; Adenovirus = 35,937.*  *^b^Poor identity match due to low quality sequencing data from the small region that did mutual build for nasopharyngeal and nasal antigen samples.*  *^c^Only compared the hemagglutinin segment of influenza viruses.*  *^d^All adenovirus discrepancies occurred between bases 17598 and 17647, in a poor quality region.* | | | |  |
