# Supplementary figures and images for: Opportunities for Enhanced Public Health Surveillance via Molecular Detection and Sequencing of Diverse Respiratory Viruses From Self-collected SARS-CoV-2 Antigen Test Swabs
Source: Open Forum Infect Dis. 2024 Aug 10;11(8):ofae447. doi: 10.1093/ofid/ofae447 (PMC11339864; doi:10.1093/ofid/ofae447)

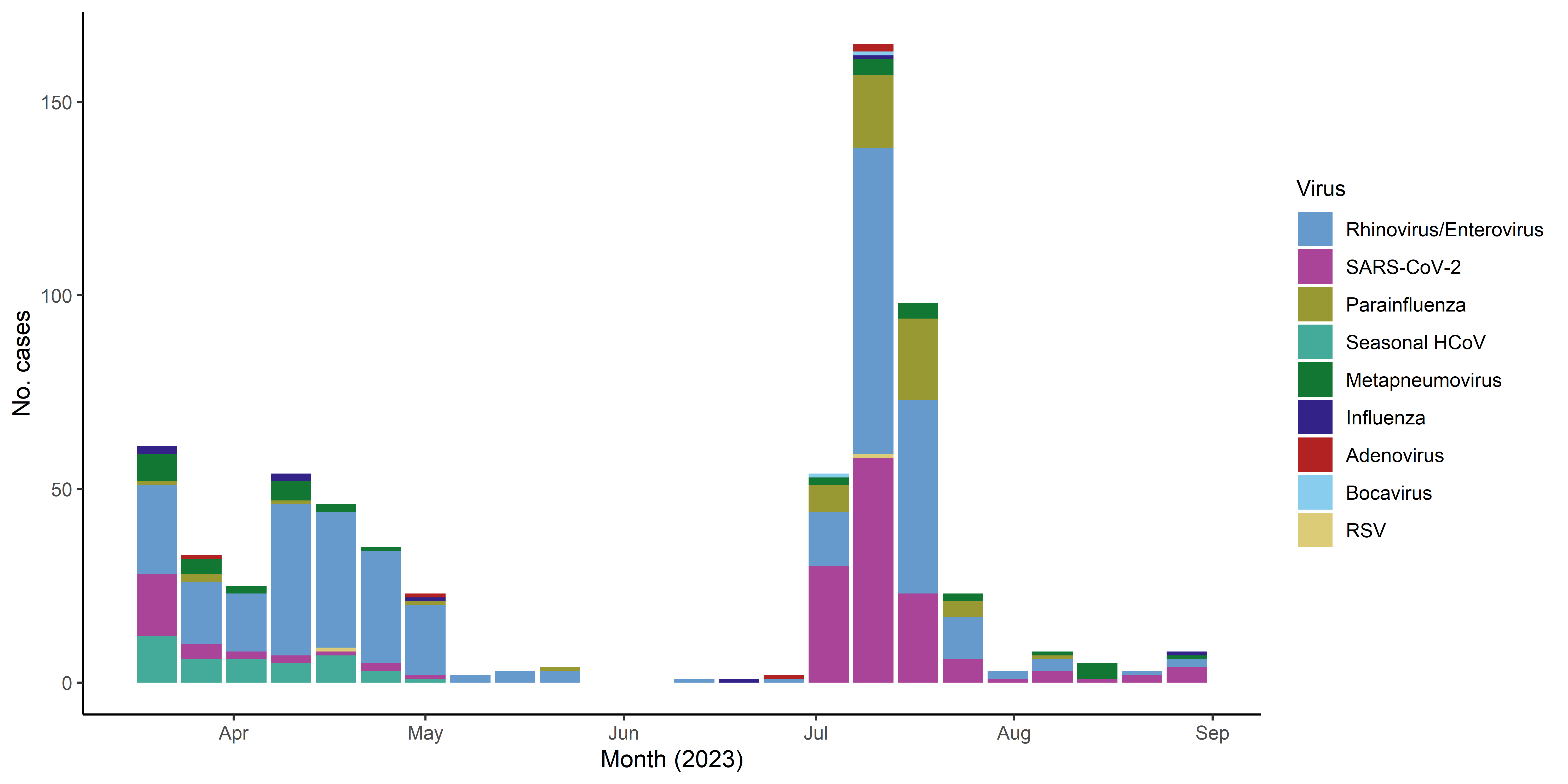

Supplement: ofae447_Supplementary_Data [file ofae447_supplementary_data.zip › Schmidt_Colombo_ARIA_FigS2.tiff]
